# Probing Enzymatic Acetylation Events in Real Time With NMR Spectroscopy: Insights Into Acyl‐Cofactor Dependent p300 Modification of Histone H4

**DOI:** 10.1002/prot.26848

**Published:** 2025-06-01

**Authors:** Sophia M. Dewing, Scott A. Showalter

**Affiliations:** ^1^ Center for Eukaryotic Gene Regulation, Department of Biochemistry and Molecular Biology The Pennsylvania State University University Park Pennsylvania USA; ^2^ Department of Chemistry The Pennsylvania State University University Park Pennsylvania USA

**Keywords:** acetylation, acylation, histone H4, nuclear magnetic resonance, p300‐CBP protein, propionylation

## Abstract

Lysine acylation is a rapidly expanding class of post‐translational modifications with largely unexplored functional roles; the study of acylations beyond acetylation is especially impeded by limited methods for their preparation, detection, and characterization in vitro. We previously reported a nuclear magnetic resonance (NMR)‐based approach to monitor Nε‐lysine acetylation following Ada2/Gcn5‐catalyzed installation of a ^13^C‐acetyl probe on the histone H3 tail. Building on this foundation, here we expand those techniques by demonstrating the installation and ^1^H, ^13^C‐HSQC based NMR detection of both ^13^C‐acetyl and ^13^C‐propionyl probes on the histone H4 tail using a mutant p300 lysine acetyltransferase (KAT) enzyme with enhanced activity. Additionally, we introduce a continuous evaluation method for acyltransferase reaction data, enabling the extraction of relative rate constants—a technique inspired by our laboratory's recent work on NMR methyltransferase kinetics. This study demonstrates that our NMR‐based approach to assay enzymatic ^13^C‐acylation is adaptable, providing a versatile platform for investigating a range of acylations, KAT enzymes, and protein substrates. Notably, in the process of developing these methods, we observed that p300 KAT may display distinct modification site preferences and regulatory mechanisms depending on the acyl cofactor utilized, underscoring the method's potential to advance the emerging field of lysine acylation biochemistry.

## 1 Introduction

While lysine acetyltransferase and deacetylase enzymes are commonly discussed in the specific context of acetyl transfer, many of these enzymes can also accommodate other acyl‐CoA cofactors and catalyze corresponding acylation reactions, broadening their functional scope [1]. Like acetylation, short‐chain lysine acylations (Kacyl) on histones are recognized by specific reader domains and are intricately linked to cellular metabolism and gene regulation [2, 3, 4, 5, 6]. Kacyl modifications also occur on non‐histone proteins, but their prevalence, biological implications, and regulatory mechanisms are still being explored [7]. Identified Kacyl modifications currently include three classes that can be organized by the chemical nature of their acyl moiety: hydrophobic groups (propionylation, butyrylation, and crotonylation), polar groups (2‐hydroxyisobutyrylation, β‐hydroxybutyrylation), and acidic groups (malonylation, succinylation, and glutarylation) [1]. This emerging understanding of Kacyl diversity and function highlights the need for expanded biochemical approaches to further elucidate their roles and regulatory mechanisms in cellular biology.

The lysine acetyltransferase p300, along with its homolog CBP (CREB‐binding protein), is widely recognized as one of the most prolific acetyltransferase enzymes due to its broad substrate specificity and robust activity [8]. p300/CBP primarily catalyze the acetylation of lysine residues on histone proteins, leading to chromatin relaxation and promoting transcriptional activation [9]. However, their role extends far beyond histone modification as p300/CBP acetylate a wide range of non‐histone proteins as well, including transcription factors and nuclear receptors, positioning them as crucial integrators of the signaling pathways and gene expression networks that regulate diverse cellular processes including DNA repair, cell cycle progression, apoptosis, and immune response [8, 10, 11, 12, 13, 14]. The broad substrate range of p300/CBP is attributed to their ability to interact with diverse partner proteins through divergent surfaces on their numerous ordered and disordered domains [15, 16]. p300's adaptability and extensive substrate catalog make it one of the most functionally diverse acetyltransferases [8].

Beyond acetylation, p300 is also known to facilitate the addition of other hydrophobic Kacyl modifications, which can impart distinct effects on gene expression [2]. For instance, lysine crotonylation, butyrylation, and propionylation have been associated with more transcriptionally active chromatin states than acetylation and shown to favor interaction with different reader proteins, suggesting that different acyl modifications might carry unique signaling information [17, 18]. This versatility in substrate and acyl‐cofactor usage underscores p300's role as a master regulator, capable of modulating diverse cellular pathways through a variety post‐translational modifications [19]. Prolific activity towards diverse protein substrates and the ability to accommodate longer chain hydrophobic acyl cofactors position p300 as the ideal enzyme for preparation of acylated protein samples for in vitro experiments.

The acetyltransferase activity of p300 is regulated by its disordered autoinhibitory loop (AIL) region (residues 1499–1560 of the human protein) [20]. Activation of p300 towards other substrates requires prior acetylation of lysine residues within the AIL, which contains 10 of p300's 13 acetylated lysine sites [20]. Structurally, the acetylation of these lysine residues activates the enzyme by changing the conformational ensemble of the AIL. Specifically, acetylation appears to increase the average distance between the AIL and the substrate binding groove due to disruption of electrostatic interactions through charge neutralization [21].

In this study, we leverage p300 KAT to achieve uniform acylation of the histone H4 tail at lysine residues K5, K8, K12, K16, and K20, using ^13^C acetyl‐CoA or ^13^C propionyl‐CoA as cofactors, followed by acyllysine detection via ^1^H, ^13^C‐HSQC NMR spectroscopy. The primary advantage of this method is that it incorporates isotopic enrichment into the acyl‐cofactors, circumventing the need for enrichment of the peptide substrate, and allowing for signal detection within a few minutes of each reaction initiation. Additionally, we introduce a continuous acyltransferase reaction assay, capitalizing on the short experiment time of the ^1^H, ^13^C‐HSQC and enabling the extraction and comparison of relative rate constants. This assay allowed us to quantify the enhancement in p300's acetyltransferase activity following truncation of its autoinhibitory loop (AIL) and to observe distinct effects of the AIL on p300's propionyltransferase activity versus acetyltransferase activity, suggesting that p300's activity may be differentially regulated depending on the acyl‐cofactor. Together, these findings underscore the potential of these NMR‐based workflows to advance the growing field of lysine acylation research.

## 2 Materials and Methods

### 2.1 Plasmids and Construct Generation

p300 KAT was originally obtained from pETduet+p300 KAT, a gift from Michael Rosen (Addgene #157793), which co‐expresses human p300 (1284–1664) with yeast Sirt2 [22]. Human p300(1284–1669) was inserted into pET His6 TEV LIC vector 1 M from Scott Gradia (Addgene #29656) with ligation independent cloning. This new p300 plasmid demonstrated improved expression. The AIL truncation p300Δ (1523–1555) was accomplished with Q5 site directed mutagenesis (NEB E0554S). p300Δ demonstrated enhanced acetyltransferase activity compared to p300 (vide infra). Both of the novel p300 expression plasmids are available through Addgene (p300, Addgene #233587, p300Δ, Addgene #233588). Human histone H4 (1–25)W was inserted into pET His6 TEV LIC cloning vector 1 M (Addgene #29656) with ligation independent cloning as has been previously reported [23]. The C‐terminal exogenous W26, inserted through Q5 site directed mutagenesis (NEB E0554S), was added to enable visualization by 2,2,2‐Trichloroethanol (TCE) stain (BeanTown Chemical 216 535) during purification.

### 2.2 Recombinant Protein Expression

All p300 KAT constructs were expressed with the same method. Transformed BL21 Rosetta *Escherichia coli* were grown in Terrific Broth (TB) supplemented with 0.4% w/v glycerol, 1 mM MgCl_2_, 100 μg/mL Ampicillin, and 25 μg/mL Chloramphenicol at 37°C to OD_600_ of 0.8–1.0. The growth was transferred to 18°C for 1 h before expression was induced with 0.5 mM IPTG for 16–18 h at 18°C. Cells were harvested by spin centrifugation and washed in 20 mM Tris pH 7.5, 20 mM sodium chloride, 2 mM EDTA before storage of the cell pellet at −80°C.

Histone H4 (1–25)W was expressed as a fusion to 6× His tagged MBP. Transformed BL21 DE3 *E. coli* were grown in Luria Broth (LB) supplemented with 50 μg/mL Kanamycin at 37°C to an OD_600_ of 0.8–1.0, and expression was induced with 1 mM IPTG for 3 h at 37°C. Cells were harvested by spin centrifugation and washed in 20 mM Tris pH 7.5, 20 mM sodium chloride, and 2 mM EDTA before storage of the cell pellet at −80°C.

### 2.3 Recombinant Protein Purification

All p300 KAT constructs were purified with the same method. All purification steps were performed on ice or at 4°C. A cell pellet was thawed at 4°C for 30 min then resuspended in 50 mM HEPES pH 7.0, 500 mM sodium chloride, 30 mM imidazole, 10% w/v glycerol, 5 mM β‐mercaptoethanol, 1 mM PMSF, 1× protease inhibitor cocktail set V (Millipore Sigma 539 137‐10VL). Cells were lysed by sonication and the lysate was clarified by spin centrifugation for 45 min at 20 000× g and 4°C. The soluble fraction was loaded onto NiNTA resin (G‐Biosciences 786–407) equilibrated with 50 mM HEPES pH 7.0, 500 mM sodium chloride, 30 mM imidazole, 10% w/v glycerol, 5 mM β‐mercaptoethanol. Bound protein was washed with 5 CV 50 mM HEPES pH 7.0, 1 M sodium chloride, 30 mM imidazole, 10% w/v glycerol, 5 mM β‐mercaptoethanol followed by 5 CV 50 mM HEPES pH 7.0, 150 mM sodium chloride, 30 mM imidazole, 10% w/v glycerol, 5 mM β‐mercaptoethanol and then eluted with 5 CV 50 mM HEPES pH 7.0, 150 mM sodium chloride, 300 mM imidazole, 10% w/v glycerol, 5 mM β‐mercaptoethanol. The 9× His or 6× His‐MBP tag was cleaved overnight at 4°C with TEV protease and dialyzed in 3.5 MWCO membrane tubing (Fischer Scientific 08‐670‐5B) against 50 mM HEPES pH 7.0, 150 mM sodium chloride, 30 mM imidazole, 10% w/v glycerol, 5 mM β‐mercaptoethanol. Dialysate was loaded onto NiNTA resin equilibrated with dialysis buffer, and flowthrough was chased with 2 CV dialysis buffer. Bound protein was eluted with dialysis buffer supplemented with 300 mM imidazole. The flowthrough was dialyzed overnight at 4°C in 3 MWCO membrane tubing against 50 mM HEPES pH 7.0, 100 mM sodium chloride, 10% w/v glycerol, 1 mM DTT. The dialysate was concentrated with a 3 MWCO spin concentrator (Millipore Sigma UFC9003) at 3500× g, 4°C so that the final concentration of p300 KAT was 200 μM, then stored at −80°C.

The histone H4 (1‐25)W cell pellet was thawed at room temperature for 20 min, then resuspended in 50 mM Tris pH 7.5, 500 mM sodium chloride, 20 mM imidazole, 2 mM β‐mercaptoethanol, and 1× protease inhibitor cocktail set V (Millipore Sigma 539 137‐10VL). Cells were lysed by sonication, and the lysate was clarified by spin centrifugation for 30 m at 14 000× g and 4°C. The soluble fraction was loaded onto NiNTA resin equilibrated with 50 mM Tris pH 7.5, 500 mM sodium chloride, 20 mM imidazole, and 2 mM β‐mercaptoethanol. Bound protein was washed with 5 CV 50 mM Tris pH 7.5, 200 mM sodium chloride, 20 mM imidazole, and 2 mM β‐mercaptoethanol and eluted with 5 CV 50 mM Tris pH 7.5, 200 mM sodium chloride, 200 mM imidazole, and 2 mM β‐mercaptoethanol. The fusion tag was cleaved overnight at 4°C with TEV (Tobacco Etch Virus) protease (a gift from Song Tan) and dialyzed overnight in 1 kDa molecular weight cutoff (MWCO) membrane tubing (Spectrum Labs 132 638) against 50 mM Tris pH 7.5, 100 mM sodium chloride, and 1 mM dithiothreitol. Dialysate was loaded onto SP Sepharose Fast Flow resin (Cytiva 17 072 910) equilibrated with dialysis buffer. Bound protein was sequentially eluted with a step gradient of 4 CV fractions containing 50 mM Tris pH 7.5 and 400, 500, 700, and 1000 mM sodium chloride. The 700 mM salt fraction was dialyzed overnight at 4°C in 1 MWCO membrane tubing against 50 mM K_2_HPO_4_ pH 7.2 and 150 mM potassium chloride. The peptide was concentrated to 2 mM with 1 MWCO spin concentrators (Cytiva MAP001C36 and MCP001C41) at 4000× g, 16°C, then stored at −80°C.

### 2.4 Reagent and Sample Preparation

CoA lithium salt (1 M equivalent, CoALA Biosciences AC02) and 1,1′,2,2′‐^13^C acetic anhydride (1.8 M equivalents, Cambridge Isotope Laboratories CLM‐1161‐1) were combined in 200 μL 0.5 M sodium bicarbonate in an Eppendorf tube and incubated in an ice bath for 45 min [24]. The product was characterized by 1D ^1^H NMR and stored at −80°C without further purification. A 200 μL reaction yielded approximately 13.4 mg of ^13^C acetyl CoA. Natural abundance acetyl‐CoA was prepared in the same way, but using ^12^C acetic anhydride (1.8 M equivalents, Sigma‐Aldrich 242 845).

To prepare proponyl‐CoA, CoA lithium salt (1 M equivalent, CoALA Biosciences AC02) and ^13^C_6_ propionic anhydride (1.6 M equivalents, Cambridge Isotope Laboratories CLM‐7844‐PK) were combined in 200 μL 0.5 M sodium bicarbonate in an Eppendorf tube and incubated in an ice bath for 60 min. The product was characterized by 1D ^1^H NMR and stored at −80°C without further purification. Natural abundance propionyl‐CoA was prepared in the same way but using ^12^C propionic anhydride (1.6 M equivalents, Millipore Sigma 240 311).

To authenticate the products of acyl‐CoA syntheses, ^1^H‐1D NMR spectra were collected with water suppression using excitation sculpting with gradients for ^12^C labeled acyl‐CoAs [25], or ^1^H‐1D with garp decoupling applied on the ^13^C channel for ^13^C labeled acyl‐CoAs. Spectra were collected on a Bruker Avance AVIII 500 MHz NMR spectrometer with TCI Cryoprobe using 65 000 data points, 16 scans, a sweep width of 16 ppm, and a spectral center of 4.69 ppm.

p300 autoacetylation reactions were conducted in 50 μL final volume, containing 200 μM p300, 2 mM ^12^C acetyl‐CoA, 50 mM Tris/50 mM BisTris/100 mM sodium acetate pH 7.5, and 1 mM DTT. The reaction was allowed to proceed for 2 h at room temperature before buffer exchange of the product back into 50 mM HEPES pH 7.0, 100 mM sodium chloride, 10% w/v glycerol, and 1 mM DTT with a 3 MWCO spin concentrator (Amicon UCF9003) at 3500× g, 4°C. The native p300 sample used for comparison was treated similarly but with dummy reaction conditions (no ^12^C acetyl‐CoA).

### 2.5 Mass Spectrometry

Single timepoint MALDI‐MS experiments were conducted by preparing 30 μL reactions containing 500 μM histone H4(1‐25)W, 5 μM KAT enzyme (p300 or p300∆), 50 mM Tris/50 mM BisTris/100 mM sodium acetate pH 7.5, and 1 mM DTT set up at room temperature. Two molar equivalents (based on the number of available lysine residues) of cofactor (acetyl or propionyl‐CoA) were added last. For example, the histone H4 tail has 5 lysine residues, so 5 mM acyl‐CoA was added to initiate the reaction. At the desired timepoint, a 5 μL sample was transferred to 45 μL MQH_2_O and heat inactivated for 5 min at 90°C. Samples were desalted with C18 spin columns (G‐Biosciences 786–930) according to manufacturer instructions. The eluent in 70% acetonitrile was dried for 3 h in a tabletop vacuum dryer and then resuspended in 5 μL chromatography grade water (Fischer L‐13780). Samples were mixed 1:1 with 20 mg/mL Super‐DHB matrix (Sigma‐Aldrich 50 862‐1G‐F) in 50% acetonitrile, 1% trifluoroacetic acid, and 1% phosphoric acid.

MALDI‐MS time courses were conducted by preparing 60 μL reactions containing 500 μM histone H4(1–25)W, 5 μM p300 or p300∆, 50 mM Tris/50 mM BisTris/100 mM sodium acetate pH 7.5, and 1 mM DTT at room temperature. 5 mM acetyl or propionyl‐CoA were spiked in at the start of the time course. At each time point, 5 μL samples were transferred to 45 μL MQH_2_O and heat inactivated for 5 min at 90°C. Samples were desalted with C18 spin columns (G‐Biosciences 786–930) according to manufacturer instructions. The eluent in 70% acetonitrile was dried for 3 h in a tabletop vacuum dryer then resuspended in 5 μL chromatography‐grade water (Fischer L‐13780). Samples were mixed 1:1 with 20 mg/mL Super‐DHB matrix (Sigma‐Aldrich 50 862‐1G‐F) in 50% acetonitrile, 1% trifluoroacetic acid, and 1% phosphoric acid.

Peptide samples were evaluated on a Bruker Ultraflextreme MALDI TOF‐TOF instrument equipped with a 355 nm frequency‐tripled NdYAG smartbeam‐II laser. The mass spectra were acquired using a factory‐configured instrument method for reflector positive‐ion detection over the 700–3500 *m/z* range. Laser power attenuation and pulsed ion extraction time were optimized to achieve the best signal‐to‐noise ratio. The instrument was calibrated with a bovine serum albumin tryptic peptide mixture (Protea, p/n PS‐204‐1). Mass spectra were opened in FlexAnalysis, smoothed (SavitzkyGolay, 0.2 *m/z*, 1 cycle), baseline‐subtracted (TopHat), and the mass lists were generated using a Snap peak detection algorithm with a signal‐to‐noise threshold set at 6 and using the Averagine SNAP average composition. Data normalization and plotting was accomplished in R‐Studio (1.4.11).

### 2.6 Circular Dichroism Spectroscopy

p300 or p300Δ were buffer exchanged into 20 mM sodium phosphate pH 7.5, 100 mM NaCl, 1 mM TCEP (VWR K831‐10G) using a 3 MWCO spin concentrator (Millipore Sigma UFC9003) at 3500× g, 4°C. Each sample was diluted to a final concentration of 0.25 mg/mL.

Circular dichroism measurements were performed on a JASCO J‐1500 spectrometer, equipped with a Peltier model PTC‐517 thermostat cell holder. Signals were recorded from 260 nm to 185 nm with a scan speed of 50 nm/min^−1^ and a band width of 1 nm at 25°C. For all experiments, data pitch was 1 nm, DIT was 4 s. The pathlength of the quartz cell was 1 mm and the p300 enzyme concentration was 0.25 mg/mL. A buffer blank was run prior to the sample for baseline subtraction and converted to units of molar ellipticity. Data was analyzed both with the Jasco Multivariate SSE program and BestSel single spectra analysis and fold recognition software.

### 2.7 NMR Spectroscopy

NMR acylation time courses were conducted by preparing 550 μL reactions (including acyl‐CoA volume) containing 500 μM histone H4 (1‐25)W, 5 μM p300 or p300∆AIL, 50 mM Tris/50 mM BisTris/100 mM sodium acetate pH 7.5, 1 mM DTT, and 5% D_2_O, which were set up at room temperature in a 5 mm NMR tube. 5 mM acetyl or propionyl‐CoA was aliquoted separately for spike in at the start of the time course.

All NMR experiments were conducted on a Bruker AVIII‐500 MHz spectrometer equipped with a triple resonance TCI single‐axis gradient cryoprobe. Samples were supplemented with 5% D_2_O and transferred to a 5 mm NMR tube. Single ^1^H,^13^C‐HSQC experiments were collected using the pulse program hsqcetgpsi from the Bruker Topspin library with a matrix size of 1024(H) × 128(C), spectral width of 5.9 × 70 ppm, eight scans per increment, and a recycle delay of 1 s.

NMR time courses were composed of consecutive ^1^H, ^13^C‐HSQC experiments collected using the pulse program hsqcetgpsi from the Bruker Topspin library with a matrix size of 1024(H) × 64(C), spectral width of 9.8 × 70 ppm, two scans per increment, and a recycle delay of 1 s. The first experiment was collected on the reaction contents before adding the acyl‐CoA cofactor to initiate reaction progress. Although ultimately omitted from kinetic analysis, experiment 1 was used for sample‐specific setup (tuning/matching, shim, and 90°^1^H pulse length) which were applied to subsequent experiments. Following the collection of experiment 1, the acyl‐CoA cofactor was mixed with the reaction contents in an Eppendorf tube, and the sample was returned to the magnet for serial collection of experiments 2 through *n*. The exact time (deadtime) was recorded between mixing of the cofactor and the start of experiment 2 and was accounted for in subsequent kinetic analysis by incorporating the deadtime into the timepoint list generated according to experiment lengths (see further details below).

For the time course analysis, phase correction and selection of a window for calculation of 1D projections was performed in Topspin 4.4 using the protocol below, which was designed to automate the process. Following 2D Fourier transformation, the final spectrum from the time series was used to pick the peak of interest (POI) in manual mode. Zero‐order phase correction was applied to the direct‐detect (^1^H) dimension, being sure to evaluate that the F2 index in the data matrix did not change for the POI following phase correction. Next, we selected the narrowest F2 index boundaries that include the column(s) for the POI, while accounting for foreseeable overlap with nearby peaks (624–628/1.84–1.89 ppm for Ac, 573–577/2.38–2.34 ppm for Pr). Finally, identical phase correction was applied to the first spectrum in the time series.

Analysis was completed using an in‐house Python script to apply uniform phase correction, baseline correction, Fourier transform, and calculation of one‐dimensional (1D) projections from the 2D data for each time point. These 1D projections were exported as text files containing intensity versus ^13^C chemical shift, and an in‐house Python script was used to determine the maximum intensity for each experiment provided as a list. A time point list (in minutes) was generated based on the deadtime and experiment lengths. Experiment 1 was excluded from the fit as the reaction was initiated at experiment 2 (time of experiment 2 = deadtime, time of experiment 3 = deadtime + experiment 2 length). Potential spectrometer deadtime between serial experiments due to software delays in queue execution or data handling latency was not accounted for when generating the time point list. All Python scripts needed to run a similar analysis within Topspin 4 and Spyder 6 are available at https://github.com/smdewing/AcNMRkinetics.

### 2.8 Data Fitting for NMR Time Series

Histone H4 acylation reactions were monitored through serial acquisition of ^1^H, ^13^C‐HSQC spectra, and the intensity of the resonance corresponding to the transferred acyl group was recorded as described. These values were exported to an excel file as intensity versus time data and fitted to the models for acylation kinetics described below. First, all data sets were fit to a mono‐exponential growth model:

(1) $$I\left(t\right)=I_{\max}∙\left(1-e^{-\mathrm{kt}}\right)$$

where the fitted parameters are *I*_max_, the asymptotic maximum intensity, and *k*, the relative rate constant. In all cases, there were systematic errors seen in the residuals from the mono‐exponential fit, resulting in the testing of two additional models to improve the quality of the fits. For data sets that demonstrate bi‐phasic exponential growth, a bi‐exponential model was used:

(2) $$I\left(t\right)=A_{1}∙\left(1-e^{-k_{1}t}\right)+A_{2}∙\left(1-e^{-k_{2}t}\right)$$

where the fitted parameters are *A*_1_ and *A*_2_, the amplitude of each exponential component; *k*_1_ and *k*_2_, the relative rate constants of each exponential component. Finally, for data sets that include a lag before the onset of exponential signal growth, as indicated by skewed positive residuals at early time points, a lagged mono‐exponential model was used:

(3) $$I\left(t\right)=A∙\left(1-e^{-\mathrm{kt}}\right)∙\frac{1}{1+e^{-\alpha \left(t-t_{o}\right)}}+C$$

where fitted parameters are *A*, the amplitude of the exponential component; *k*, the relative rate constant of the exponential component; *t*_0,_ the transition time between the initial and exponential phase; slope around *t*_0_ (*α*); and *C*, the initial amplitude of the lagged phase.

## 3 Results

### 3.1 Generation of a Robust p300 KAT Enzyme Construct

**FIGURE 1:**
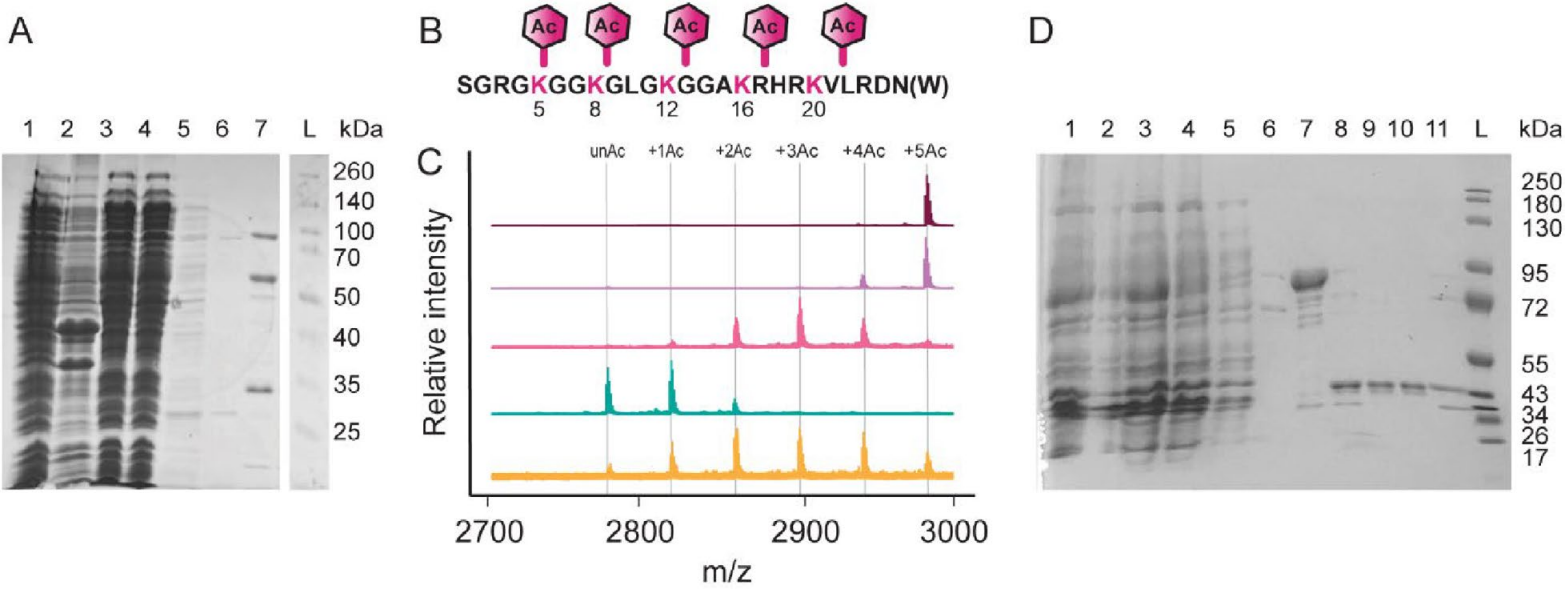
Generation of a p300 construct with improved yield. (A) Representative expression and purification gel of p300 KAT from pETduet+p300 KAT suggests a total yield of ~1 mg/L of media. Lanes show (1) Whole cell lysate (2) Insoluble pellet (3) Soluble supernatant (4) NiNTA flowthrough (5) Low salt (150 mM) wash (6) High salt (1 M) wash (7) NiNTA elution alongside the protein standard ladder lane (L) (ThermoFischer 26 634). p300 KAT is the faint band ~50 kDa in lane 7. (B) Schematic representation of the histone H4 (1‐25)W construct used in subsequent experiments. This construct can be uniformly acetylated by p300 KAT on each of its five available lysine sites. (C) Demonstration of variable activity between p300 KAT preparations shown by MALDI‐MS spectra of identical acetylation reactions using presumably equivalent enzyme preparations. (D) Representative expression and purification gel for p300 KAT from pET His6 MBP TEV p300(1284–1669) LIC (Addgene #233587). Total yield was typically > 10 mg/L of media. Lanes show (1) Whole cell lysate (2) Insoluble pellet (3) Soluble supernatant (4) NiNTA flowthrough 1 (5) Low salt (150 mM) wash (6) High salt (1 M) wash (7) NiNTA elution 1 (8) TEV protease dialysate (9) NiNTA flowthrough 2 (10) NiNTA wash 2 (11) NiNTA elution 2 (12) Protein ladder (NEB P7719S). Cleaved p300 KAT is the band ~43 kDa in lanes 9 and 10 (expected molecular weight once cleaved is 44 684 Da). Note this is very close to the expected molecular weight of 41 584 Da for the cleaved MBP solubility tag.

These experiments were initiated with p300 KAT from a commercially available pET‐DUET vector that co‐expresses human p300(1286–1664) as a His tagged fusion protein along with yeast Sir2(87–562) (Addgene #157793). This plasmid yielded poor expression of p300 KAT (expected mass of 45 994 Da) and no observable expression of Sir2 (expected mass of 53 838 Da) (Figure 1A). Attempts to further improve purity with additional chromatography steps resulted in lower enzyme activity. Additionally, expression and purification from this system resulted in widely variable enzyme activity across different preparations when we attempted to uniformly acetylate the five available lysine residues in histone H4 (1–25)W. Representative mass spectra display variable reaction products from identical acetylation reactions using four different expressions and purifications of p300 KAT (Figure 1B,C). To improve the reproducibility of acetylation activity across p300 KAT preparations, we generated an alternative p300 KAT construct by subcloning p300 (1284–1669) into pET His6 MBP LIC. The resulting plasmid is available on Addgene (p300 KAT LIC 1 M, Addgene #233587). *E. coli* transformed with this construct grew more quickly and reliably than with the previously available expression system, while the expression level and purified yield was improved at least 10‐fold (> 10 mg/L compared to < 1 mg/L) (Figure 1D). While expression from the LIC vector significantly improved enzyme yield, challenges remained in achieving uniform acetylation of the H4 tail, particularly in catalyzing the fifth acetylation event.

### 3.2 p300 Auto‐Inhibitory Loop Truncation Increases Acetyltransferase Activity

**FIGURE 2:**
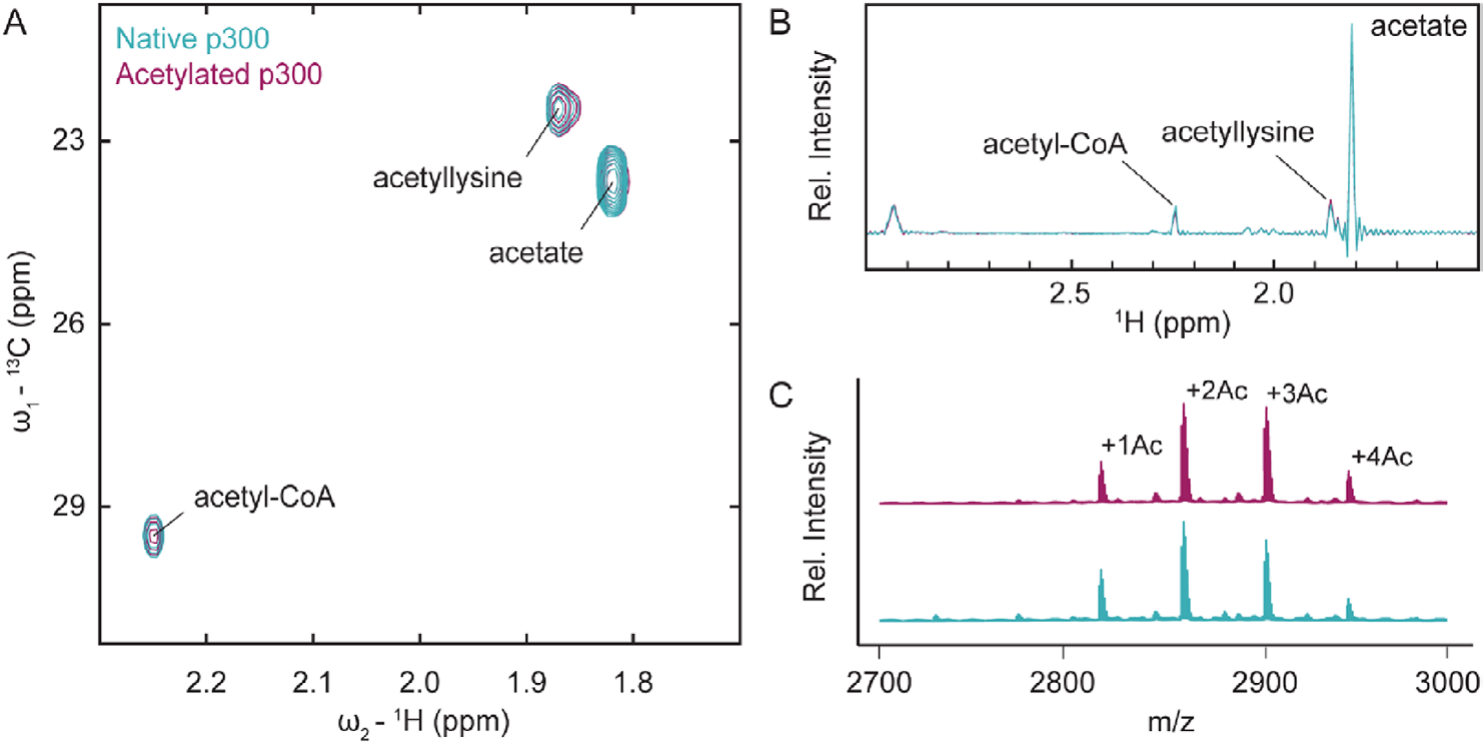
Pre‐acetylation of p300 does not meaningfully alter acetyltransferase activity. (A) Overlaid ^13^C, ^1^H‐HSQC NMR spectra showing reaction products after 1 h treatment of histone H4 (1‐25)W with native p300 (teal) or p300 prescribed to a 2‐h incubation with acetyl‐CoA (purple). (B) overlaid 1D projections of the ^13^C, ^1^H‐HSQC experiments demonstrate nearly equivalent peak heights for the acetyl‐CoA (2.25 ppm) and acetyllysine (1.87 ppm ^1^H) products. (C) MALDI‐MS spectra showing the distributions of reaction products.

Given that p300's acetyltransferase activity is regulated by acetylation of its AIL, and cellular lysine acetylation levels are constrained by acetyl‐CoA availability, we hypothesized that sub‐optimal p300 KAT activity could be attributed to insufficient acetylation of the AIL during expression. In the initial manuscript identifying and characterizing the AIL, Thompson et al. [20] demonstrated that prescribing their recombinant p300 KAT preparations (generated alongside Sir2) to an autoacetylation treatment period (2‐h incubation with acetyl‐CoA), prior to introduction of substrate, increased its enzymatic activity approximately fourfold. We tried adopting this autoacetylation step as a quality control measure, but no meaningful difference in acetyltransferase activity was observed by MS or NMR following the two‐hour acetyl‐CoA incubation (Figure 2). Therefore, we conclude that, in the absence of Sir2 co‐expression, the AIL is sufficiently acetylated during expression to support activity.

**FIGURE 3:**
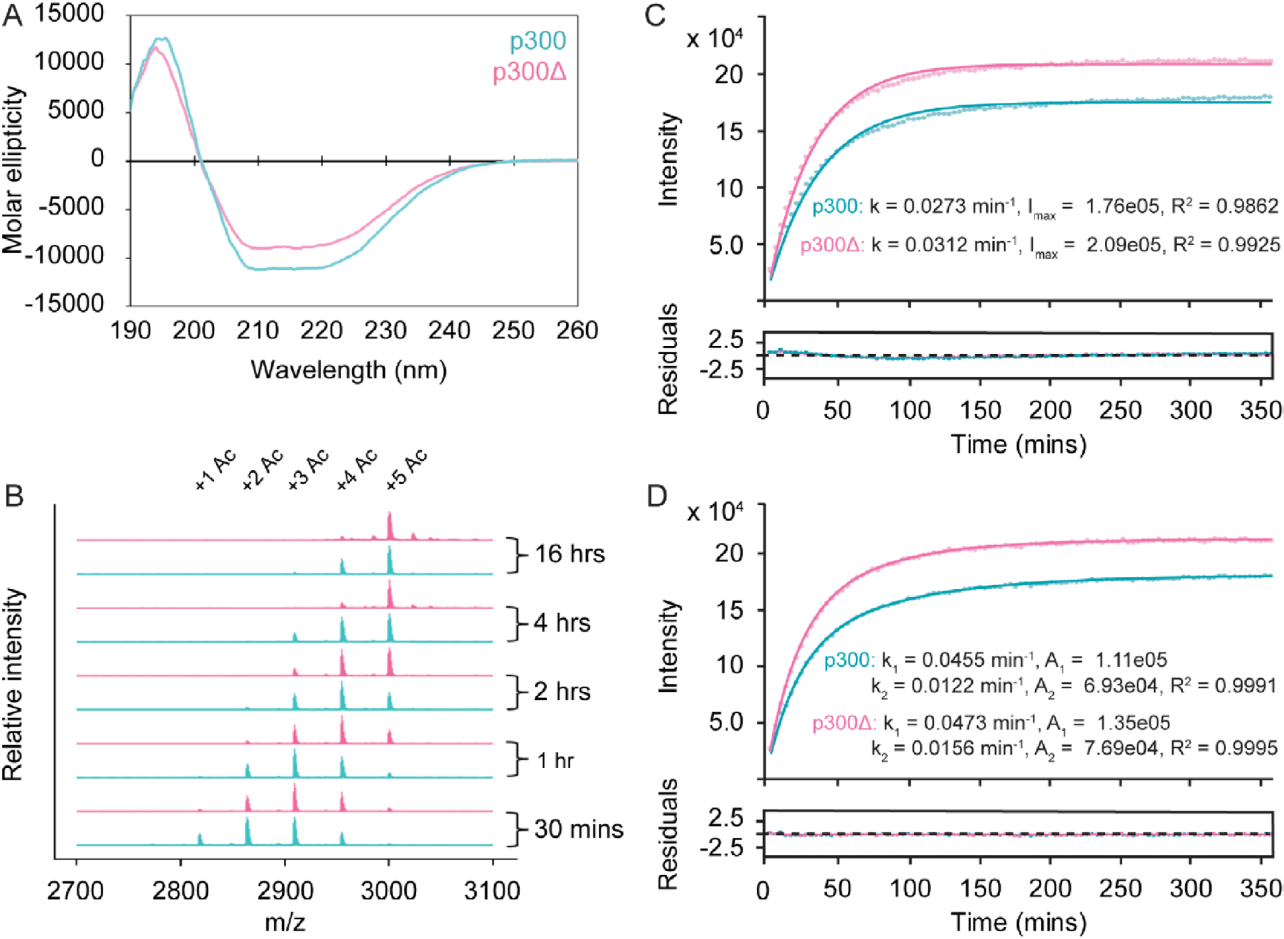
Comparison of p300 and p300Δ acetyltransferase activity towards the histone H4 tail. (A) Circular dichroism spectra for p300 (teal) and p300Δ (pink) used to estimate secondary structure content with BestSel. (B) MALDI‐MS time courses comparing relative acetyltransferase efficiency of p300 (teal) and p300Δ (pink) using the histone H4 tail as a substrate. (C) Mono‐exponential fit of acetyltransferase reaction curves from a ^1^H‐^13^C, HSQC based NMR time course for p300 (teal) and p300Δ (pink) acetyltransferase reactions with the histone H4 tail. Progress curves show the increase in intensity for the acetyllysine resonance centered at 1.86 ppm ^1^H, 22.1 ppm ^13^C over time, each data point represents the maximum acetyllysine peak intensity from a 3 min and 36 s experiment. Progress curves were fit with a mono‐exponential kinetic model to estimate relative turnover rates (*k*) and maximum intensity (*I*_max_). (D) Progress curves for p300 (teal) and p300Δ (pink) acetyltransferase reactions with the histone H4 tail fit with a bi‐exponential kinetic model to estimate relative turnover rates (*k*_1_ and *k*_2_) and relative contributions to the maximum intensity (*A*_1_ and *A*_2_). *R* ^2^ values are included in C and D to indicate the goodness of fit, and fit residuals for each data point are displayed at scale relative to 20% of the maximum data intensity.

We next considered whether the AIL could instead be removed to increase p300 KAT activity without compromising structural integrity or causing cellular toxicity during expression. This strategy had also been employed by Thompson et al. [20] and found to produce a constitutively active version of the enzyme. We truncated the AIL through deletion of residues 1523–1555, preserving innocuous amino acids at the N‐ and C‐termini of the AIL to maintain structural integrity while eliminating most of the lysine residues responsible for establishing inhibitory interactions with the substrate binding groove [21]. The resulting plasmid is available on Addgene (p300dAIL KAT LIC 1 M, Addgene #233588). This AIL truncation mutant (p300Δ) had no observable effects on cellular growth or expression levels. We assessed the enzyme's structural integrity through circular dichroism spectroscopy and found no change in spectral qualities between p300 and p300Δ aside from a difference in signal attributable to the change in molecular weight (both samples were run at 0.25 mg/mL) (Figure 3A). Analysis with BestSel indicated no change in secondary structure content [26]. We then compared the relative activity of p300 and p300Δ by setting up identical acetylation reactions on the histone H4 tail and pulling samples for analysis by mass spectrometry at discrete timepoints (Figure 3B). This MS time course showed that p300Δ achieved homogeneous acetylation of the five available lysine residues (5Ac) on the H4 tail within 4 h, while wildtype p300 remained stuck at a mixture of 4/5Ac even at 16 h. This suggested that p300 acetyltransferase activity improved upon truncation of the AIL. However, we were interested in a more quantitative and continuous comparison of the relative enzyme efficiency.

To obtain a superior estimate of relative acetyltransferase activity, we utilized ^1^H, ^13^C‐HSQC NMR detection of acetyllysine formation, measured in real time (Figure 3C). Qualitatively, this progress curve reinforces that H4 tail acetylation saturates more quickly and to a greater extent with p300Δ compared to p300. The difference in maximum intensity between these experiments can be attributed to incomplete acetylation with p300 as observed in the MS time course (Figure 3B). Collection of continuous data in this format enabled fitting according to a first‐order exponential kinetic model to enable extraction of relative rate constants; an approach that was adapted from the NMR methyltransferase kinetics method recently reported by Usher et al. [27].

A mono‐exponential model estimates that p300 has a relative turnover rate of 0.0273 min^−1^ compared to 0.0312 min^−1^ for p300Δ under the deployed experimental conditions. Note that these reactions were performed with the substrate (500 μM H4 tail, 5 mM acetyl‐CoA) well in excess of the enzyme (5 μM) concentration, so *k* approximates *k*_cat_. However, while the *R* ^2^ values for these fits are high, the fit residuals systematically underestimate then overestimate the intensity for both samples, indicating that the behavior of the system is not fully captured by this model. Considering that there are five lysine residues available for acetylation by p300, this is perhaps unsurprising. Additionally, we previously observed that p300 has a strong preference to acetylate H4K5, K8, and K12 prior to H4K16 and K20 in the context of this system [23], providing a key experimental premise to support the proposition that p300 displays distinct kinetic properties toward these two groups of lysine residues.

Based on this hypothesis, we applied a bi‐exponential model to fit these reaction data. As can be seen in Figure 3D, the goodness of fit and the randomness of the residuals improve with this model. Additional models that attempted to incorporate more than two exponential functions failed to find solutions, reinforcing that the bi‐exponential model best captures the behavior in these progress curves. The bi‐exponential model estimates two relative turnover rates and reports the relative contribution of reach rate to the observed total intensity. According to this model, both relative turnover rates are faster for p300Δ (0.0473 and 0.0156 min^−1^) than for p300 (0.0455 and 0.0122 min^−1^). In both cases, the relative turnover rate for group 1 is 3–4× times faster than the relative turnover rate for group 2.

Notably, the relative amplitude contribution of rate 1 (the faster rate) is approximately 60% while the relative amplitude contribution of rate 2 (the slower rate) is approximately 40%, indicating that rate 1 corresponds to a group of 3 lysine residues while rate 2 corresponds to a group of 2 lysine residues. According to our previous observations, K5, K8, and K12 would then compose the group of 3 lysine residues that are preferred substrates for p300 and are acetylated at a faster rate, while K16 and K20 would compose the group of 2 lysine residues that are less preferred substrates and are acetylated at a slower rate [23]. Overall, these continuous NMR data provide a robust and quantitative estimate of the activity enhancement accomplished by truncation of p300's AIL that is in alignment with the qualitative comparison provided through the MS approach. Considering our pre‐existing knowledge of this system, we were able provide additional details that enabled deconvolution and specific assignment of the multiple rate constants contributing to the shape of these progress curves.

### 3.3 NMR‐Based Monitoring of Propionyltransferase Reactions

**FIGURE 4:**
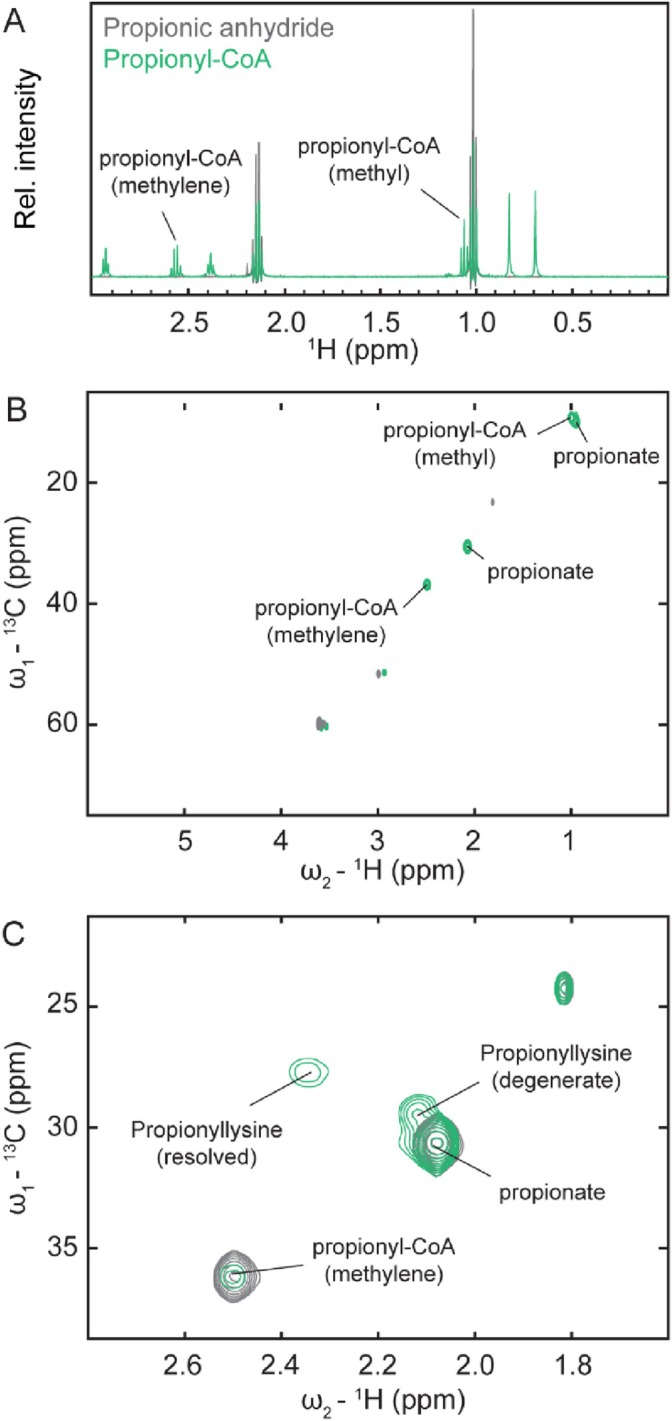
Validation of ^12^C propionyl‐CoA synthesis and control reactions to enable ^13^C propionylation resonance assignments. (A) Proton 1D NMR spectra showing the reaction product of the propionyl‐CoA (green) synthesis reaction from propionic anhydride precursor (gray). The peaks at 1.06 and 2.56 ppm, respectively, were assigned to the methyl and methylene protons of the CoA conjugated propionyl moiety based on comparison to reference proton 1D spectra provided by CoALA Biosciences (SKU PC01). (B) Overlaid ^13^C, ^1^H‐HSQC NMR spectra of the propionyltransferase reaction mixture (without enzyme) before adding ^13^C propionyl‐CoA (gray) and after adding ^13^C propionyl‐CoA (green). (C) Overlaid ^13^C, ^1^H‐HSQC NMR spectra for p300 catalyzed propionylation of the histone H4 tail (green) overlaid with the appropriate no enzyme control (gray).

We were interested in extending these methods to enable sample preparation and NMR investigation of hydrophobic acylations beyond acetylation known to be catalyzed by p300. According to Peter et al. [28] the symmetric anhydride synthesis we previously employed to make ^13^C acetyl‐CoA can be applied to prepare butyryl, succinyl, propionyl, and crotonyl‐CoAs. Synthesis of ^13^C propionyl or succinyl‐CoA was possible based on the commercial availability of the respective ^13^C symmetric anhydride precursors, and we chose to pursue ^13^C propionyl‐CoA synthesis as it is a known cofactor for p300. Prior to ^13^C synthesis, we demonstrated that we could synthesize propionyl‐CoA using ^12^C propionic anhydride precursor. The peaks at 1.06 and 2.56 ppm in the proton 1D NMR spectra correspond respectively to the methyl and methylene protons of the CoA conjugated propionyl moiety, indicating successful synthesis (Figure 4A).

We adapted the workflow used for NMR acetyltransferase kinetics but using ^13^C propionyl‐CoA as the cofactor. We confirmed the synthesis and visibility of the ^13^C propionyl‐CoA probe through the ^1^H, ^13^C‐HSQC spectrum (Figure 4B). The ^1^H, ^13^C‐HSQC of ^13^C propionyl‐CoA has two resonances attributable to the methyl and methylene protons of the CoA conjugated propionyl moiety (1.00 ppm ^1^H, 9.23 ppm ^13^C and 2.49 ppm ^1^H, 37.08 ppm ^13^C) and two resonances attributable to the methyl and methylene protons of residual ^13^C propionate from propionyl‐CoA synthesis (0.95 ppm ^1^H, 10.05 ppm ^13^C and 2.08 ppm ^1^H, 30.72 ppm ^13^C). We then validated that the propionyllysine resonance(s) would be visible and resolved in the ^1^H, ^13^C‐HSQC (Figure 4C). Upon initiation of the transfer reaction, we observed emergence of one well resolved resonance (2.35 ppm ^1^H, 27.37 ppm ^13^C) and another resonance centered at 2.12 ppm ^1^H, 29.37 ppm ^13^C that partially overlaps with the ^13^C propionate resonance centered at 2.08 ppm ^1^H, 30.72 ppm ^13^C. The resolved position of the propionyllysine resonance at 2.35 ppm ^1^H, 27.37 ppm ^13^C enables facile quantification of its signal intensity in the continuous format.

### 3.4 p300 Displays Distinct Behavior When Catalyzing Propionyltransferase Reactions

**FIGURE 5:**
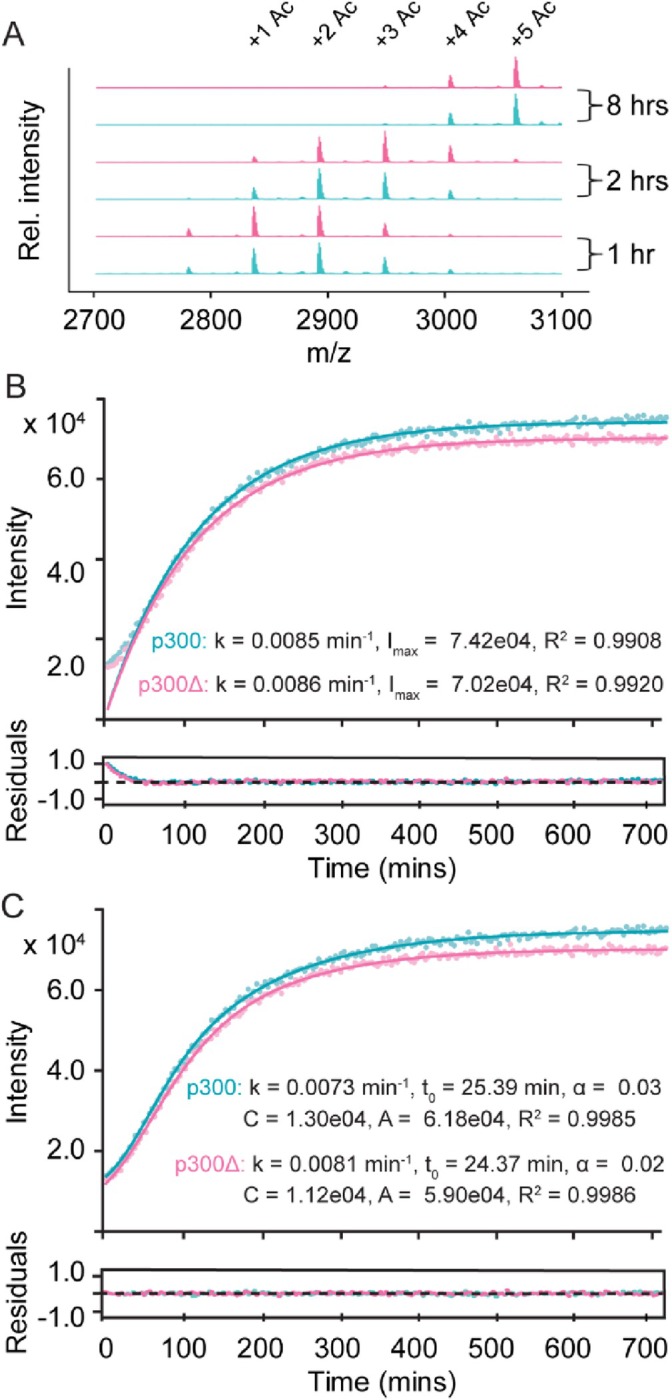
Comparison of p300 and p300Δ propionyltransferase activity towards the histone H4 tail. (A) MALDI‐MS time course comparing relative propionyltransferase efficiency of p300 (teal) and p300Δ (pink) using the histone H4 tail as a substrate. (B) Progress curves for p300 (teal) and p300Δ (pink) propionyltransferase reactions with the histone H4 tail fit with a mono‐exponential kinetic model to estimate relative turnover rates (*k*) and maximum intensity (*I*_max_). (C) Progress curves for p300 (teal) and p300Δ (pink) propionyltransferase reactions with the histone H4 tail fit with a lagged mono‐exponential kinetic model to estimate relative turnover rate (*k*_1_), amplitude (A), lag time (*t*_0_), and slope around *t*_0_ (*α*). *R* ^2^ values are included in B and C to indicate the goodness of fit, and fit residuals for each data point are displayed at scale relative to 20% of the maximum data intensity.

We evaluated the efficiency of propionyl transfer to the histone H4 tail with p300 and p300Δ in an MS time course (Figure 5A). Intriguingly, there appeared to be no observable difference in the distribution of propionylation states or extent of propionylation at these discrete timepoints, though differences in acetyltransferase efficiency had been observed by MS at equivalent timepoints using the same enzyme preparations (Figure 3B). This could indicate that while acetyltransferase activity is substantially regulated by the AIL, propionyltransferase activity is not. However, this observation could also be an experimental artifact resulting from the selected timepoints, or the semi‐quantitative nature of MS. In this case, a continuous and quantitative method was necessary to discern between these possibilities.

To further characterize propionyl transfer, we compared the relative propionyltransferase activity of p300 and p300Δ with NMR time courses (Figure 5B). In alignment with the MS time course, the relative propionylation activity between p300 and p300Δ did not appear to exhibit the same differences as acetyltransferase activity when assayed in the continuous NMR format. The mono‐exponential kinetic model estimated similar relative turnover rates of 0.0085 min^−1^ for p300 and 0.0086 min^−1^ for p300Δ, and the maximum intensity values for the two reactions were closer in magnitude than those for the acetyltransferase reactions, which is consistent with the MS results. These reactions were performed with the same conditions as the acetyltransferase reactions, with the substrate (500 μM H4 tail, 5 mM propionyl‐CoA) well in excess of the enzyme (5 μM) concentration, so k again approximates *k*_cat_. However, both transfer reactions again display patterned residuals, specifically underestimating signal intensity at early reaction timepoints, indicating that the behavior of the system is once again not fully captured with the mono‐exponential model, particularly at early timepoints.

To address the properties of these early timepoints, we introduced a lag time parameter (*t*_0_) into the mono‐exponential model to account for the transition between the early phase, where an uncharacterized process initially dominates the recorded data, and the second phase where we can monitor exponential reaction progress. This model also incorporates a baseline parameter, C, which defines the initial amplitude of the process that masks exponential growth at early timepoints. This lagged mono‐exponential model improved the goodness of fit and randomness of the fit residuals for both reactions. The estimated transition times (25.39 and 24.37 min) and relative turnover rates (0.0073 and 0.0081 min^−1^) for p300 and p300Δ remained highly similar according to this more accurate and descriptive model (Figure 5C). The application of a lagged bi‐exponential model did not improve the goodness of fit in this case, indicating that the lagged mono‐exponential model is sufficient to describe reaction behavior. Notably, these propionylation rate constants are 5–6× slower than the *k*_1_ rate constants estimated for the acetyltransferase reactions, and ~1.5× slower than the *k*_2_ rate constants, indicating that p300 generally transfers less efficiently from the propionyl‐CoA cofactor compared to the acetyl‐CoA cofactor (Figure 3D).

Compared to the difference in activity observed between the p300 and p300Δ acetyltransferase reactions, these data collectively indicate that the AIL regulates the acetyltransferase activity of p300 to a greater extent than it regulates propionyltransferase activity. Furthermore, the differences between the models that best account for the behavior of the acetyltransferase and propionyltransferase reactions indicate that p300 may employ different mechanisms for transfer from various acyl cofactors. Specifically, the propionyltransferase progress curves display a distinct lag phase, while the acetyltransferase progress curves do not. Furthermore, the exponential phase of the acetyltransferase reaction was best described by a bi‐exponential model, whereas the propionylation reaction was well described by a mono‐exponential model. This suggests that p300 may exhibit different site‐specific preferences when modifying lysine residues depending on the acyl cofactor. These observations could be explained by a difference in the binding surfaces or kinetic mechanisms employed by p300 for transfer from different acyl cofactors. However, distinguishing which mechanism(s) underlie these observations would require more in‐depth kinetic and mutational analyses.

## 4 Discussion

This study introduces an optimized workflow and reagent set for recombinant expression and purification of p300 KAT to facilitate acylation of protein substrates in vitro. Additionally, we expand on previously developed methodologies by introducing an approach for synthesizing, transferring, and detecting _13_C‐labeled propionyllysine moieties on proteins of interest via NMR spectroscopy, as well as a continuous ^1^H, ^13^C‐HSQC assay for measuring acetyltransferase and propionyltransferase reaction kinetics. These workflows offer potential for further expansion to other Kacyl modifications, contingent on the availability of suitable ^13^C‐enriched precursors.

The MALDI‐MS and NMR time‐course analyses presented here provide complementary insights into the processing of the H4 tail by p300 during acyltransferase reactions. While the MS approach effectively illustrates acylation state distributions and offers a qualitative snapshot of relative activity, the NMR approach provides a continuous and quantitative measure of activity, though without acylation state resolution. For substrates with better‐resolved acyllysine resonances, NMR could feasibly assign specific rate constants to distinct acylation sites within a single assay. For now, the combination of these analytical techniques provides detailed information on the progression of acyltransferase reactions.

The development of these methods yielded new insights into the enzymatic activity and regulatory mechanisms of p300, highlighting their intrinsic value to the advancing field of lysine acylations. Our findings suggest that the regulatory principles governing p300's acetyltransferase activity may not uniformly apply to other acylation reactions. Specifically, we observed that the AIL impacts acetyltransferase activity to a greater extent than propionyltransferase activity, and that the modification site preference and kinetic mechanism employed by p300 on the same protein substrate appears to vary in accordance with the acyl cofactor. Additional kinetic and mutational studies are warranted to elucidate the mechanisms underlying these differences. A Michaelis–Menten analysis, for instance, could help determine whether variations in substrate binding affinities distinguish acetyltransferase from propionyltransferase reactions in p300 and p300Δ, providing further insights into the regulatory mechanism of p300's AIL.

## 5 Conclusion

Lysine acylation is a critical biochemical process with implications for protein function and disease mechanisms, yet the distinct functional consequences of this diverse class of modifications remain poorly understood. This study incorporates routine NMR techniques and a novel chemical biology probe into a streamlined biochemical workflow to monitor lysine acetylation and propionylation reactions in real time, providing tools to advance our understanding of how KAT enzymes interact with various substrates and acyl cofactors, demonstrating their potential to significantly advance the emerging field of lysine acylations.

## Author Contributions

**Sophia M. Dewing:** conceptualization, investigation, writing – original draft, visualization, writing – review and editing, methodology, formal analysis, data curation. **Scott A. Showalter:** conceptualization, funding acquisition, writing – review and editing, validation, formal analysis, project administration, supervision, data curation, resources.

## Conflicts of Interest

The authors declare no conflicts of interest.

## Peer Review

The peer review history for this article is available at https://www.webofscience.com/api/gateway/wos/peer‐review/10.1002/prot.26848.

## Data Availability

The data that support the findings of this study are openly available in Penn State ScholarSphere at https://scholarsphere.psu.edu/catalog?q=showalter.
